# Perceived Psychological Feelings Make Important Contributions to the Symptoms of Common Mental Disorders of Medical Staff During the COVID-19

**DOI:** 10.3389/fpsyt.2021.738610

**Published:** 2022-01-27

**Authors:** Bing Han, Chao Ma, Zhaorui Liu, Rongmeng Jiang, Tingting Zhang, Ying Wang, Hongguang Chen, Jing Wen, Yueqin Huang

**Affiliations:** ^1^Clinical and Research Center of Infectious Diseases, Beijing Ditan Hospital, Capital Medical University, Beijing, China; ^2^Peking University Sixth Hospital, Peking University Institute of Mental Health, NHC Key Laboratory of Mental Health (Peking University), National Clinical Research Center for Mental Disorders (Peking University Sixth Hospital), Beijing, China

**Keywords:** COVID-19, medical staff, mental health, epidemiology, psychological intervention

## Abstract

**Objective:**

Lack of information about mental health status among medical staff during the epidemic of COVID-19 in China is one of the major barriers to psychological interventions. This paper aims to evaluate the contributions of perceived psychological feelings to the symptoms of common mental disorders among medical staff during the epidemic of COVID-19 in China.

**Method:**

A large sample of medical staff based on a non-probability sampling design was collected from February 17 to 24, 2020. The symptoms of common mental disorders were screened based on self-reported instruments to evaluate psychological distress, anxious symptoms, and depressive symptoms during the past week. Perceived psychological feelings were based on self-report. Logistic regressions and elastic net regularizations were used to evaluate the independent effect of the psychological feelings controlled by hospitals and participants characteristics.

**Results:**

Totally 4,677 medical staff completed the survey. The prevalence of psychological distress, anxious symptoms, and depressive symptoms were 15.9% (95% CI 14.8–16.9), 16.0% (95% CI 15.0–17.1), and 34.6% (95% CI 33.2–35.9). Feelings of having adequate personal protective equipment, receiving enough emotional supports from both family members and colleagues were significantly associated with fewer symptoms of common mental disorders, while the feelings of overloaded work and insufficient rest times contributed to more psychological problems.

**Conclusions:**

Psychological feelings make important contributions to the symptoms of common mental disorders of medical staff during the epidemic of COVID-19. Strategies of psychological aids or interventions could be developed based on these feelings.

## Introduction

The epidemic of coronavirus disease 2019 (COVID-19) mainly in Wuhan, China attracted global attentions at the beginning of 2020. From January 20, 2020, the date of the first implementation of the lockdown policy in China, to March 11, 2020 when the WHO declared COVID-19 as pandemic, the National Health Commission (NHC) of People's Republic of China reported 80,793 people from 31 provinces diagnosed with COVID-19 and 3,169 died of this infectious disease. Totally 92.3 % of patients and 66.8% of deaths were recorded during the first month after the lockdown (1).

The main routes of the transmission of COVID-19 are respiratory droplets and close contact, which leads to the fact that doctors and nurses became high-risk exposure population, especially during the beginning stage of the epidemic when these health professionals did not realize the risk of infection. Based on a report from the China-WHO COVID-19 joint expert team, 2,055 health professionals from 476 hospitals were diagnosed with COVID-19 during the first month after the lockdown (2). In that month, with the rapid increase of the number of patients, doctors and nurses had to face enormous workload and high-risk infection, which may lead to psychological problems such as anxiety or depression.

Recent studies have arisen the concerns around the negative psychological effects during the pandemic and demonstrated that the outbreak brings a greater risk of psychological distress for both medical staff and general public (3). A study carried out in in Singapore and India among 906 healthcare workers from five major hospitals found that the prevalence of depression, anxiety, and psychological distress was 5.3, 8.7, and 3.8%, respectively (4). Another study collected information from 2,042 medical staff and 257 administrative staff based on an online questionnaire and found that medical staff presented a higher prevalence of mild to moderate (22.6%) and severe (2.9%) anxiety, and mild to moderate (11.8%) and severe (0.3%) depression than non-clinical staff did (5). The above studies highlight the needs of mental health care among medical staff (6, 7).

In order to maintain healthy mental health status of doctors and nurses, the NHC of China issued a notice, pointing out the importance of providing psychological interventions and supports to medical workers. Psychological feelings are crucial to implement effective psychological intervention. Both structured and unstructured psychotherapeutic interventions, like psychodynamic therapy (8) and supportive therapy (9), have emphasized the importance of investigating and ventilating experiences and emotional feelings in understanding and interpreting the origin of long-standing mental problems. This paper aims to test the contributions of psychological feelings to the symptoms of common mental disorders among doctors and nurses in hospitals during the epidemic of COVID-19 in China. Findings from this paper may provide suggestions for the development of strategies of psychotherapies or other psychological interventions.

## Methods

### Participants

A Web-based survey using a program called Questionnaire Star was carried out to do a cross-sectional study on the symptoms of common mental disorders among doctors and nurses working at hospitals in mainland China from February 17 to 24, 2020, when the epidemic of COVID-19 was most severe in China. WeChat messages were used to reach these medical staff. Hospitals include general hospitals, traditional Chinese medicine hospitals, integrated Chinese and Western medicine hospitals, minority national hospitals, various specialized hospitals, and nursing homes, excluding specialized disease prevention and treatment hospitals, maternal and child health care hospitals, and sanatoriums (10).

The sample was obtained based on a non-probability sampling design. Doctors and nurses participated in the survey using a quick response (QR) code, which is a mobile phone readable bar code that can store website link of the questionnaire. An approach being similar as quota sampling was applied during the sample size calculation to ensure enough subjects could be recruited from each important layer. In this survey, the status of working department was defined as a layer factor. Doctors and nurses from any of the following four departments, including infection, emergency, intensive care unit, and respiratory, were defined as frontline medical staff. The rest of staff were treated as non-frontline medical staff. Frontline medical staff usually provided direct treatments or helps for COVID-19 patients and therefore had higher risk of the exposure to COVID-19 than non-frontline medical staff did.

The survey was announced among 23 small-scale WeChat groups (50 to 60 persons each) and 5 large-scale WeChat groups (500 persons each) consisted of doctors and nurses. Additional 60 department directors were asked to introduce the survey to the medical staff in their departments. After eliminating individuals with missing information on survey items, a total of 4,677 doctors and nurses who completed the online survey constituted the final sample of this survey. The objectives and procedures of this study were clearly stated at the beginning of the survey. All participants read the informed consents before voluntarily participating in the survey. All procedures in the survey complied with relevant ethical standards of the internal and national research committee on human experimentation and with the Helsinki Declaration of 1975, as revised in 2008. All procedures involving human subjects were approved by the Ethical Committee of Beijing Ditan Hospital Capital Medical University (JINGDILUNKE(2020)-(012)-01). The committee approved the exempting of written informed consent from the subjects, as the survey was anonymous.

### Main Outcome Measures

All participants were assessed with a battery of instruments that measures psychological distress, anxious symptoms, and depressive symptoms. In particular, psychological distress was evaluated by the Chinese version of WHO 20-item Self-Reporting Questionnaire (SRQ-20) (11), a self-report measurement with 20 items designed to screen for general psychiatric disturbances. SRQ-20 has been tested in a Chinese community sample with satisfied validity and reliability (12). Respondents were categorized to the group with psychological distress if their SRQ total scores were greater than seven. Anxious symptoms were assessed with the Zung Self-rating Anxiety Scale (SAS) (13), which is a 20-item self-reported questionnaire targeted at the measurement of anxious symptoms. The SAS standard score was converted into four categories, including none (<50), mild (50–59), moderate (60–69), and severe (≥70). The Zung Self-rating Depression Scale (SDS) (14) is a well-validated, 20-item self-report questionnaire that measures the level of depressive symptoms. Same scheme was applied to SDS standard score in this study. The Chinese version of SAS (15) and SDS (16) were used in this survey. Previous studies proved that the two instruments had satisfied validity (17, 18).

### Perceived Psychological Feelings

Information of perceived psychological feelings was collected based on self-report. Feelings of the following aspects were asked during the survey, including heavily overloaded work, not having enough time to rest, perceived physical and mental health conditions in the past, adequacy of protective equipment while working, and cares or supports from families, colleagues, and department directors.

### Hospitals and Participant Characteristics

Both hospitals and participants characteristics were considered to have potential correlations with the presence of the symptoms of common mental disorders and therefore were recorded in the survey. Hospital attributes included hospital classification (general or specialized), hospital level (primary and unclassified, secondary, or tertiary), and the type of hospital (designated hospital for COVID-19 patients or not). Sociodemographic features of participants were occupation (doctor or nurse), age, gender, and living condition (living with family number most of the time or not). Working status of the sample consisted information of professional titles (primary and lower, intermediate, or senior), department (high or low risk of exposure to COVID-19), training experiences of the practice guideline of COVID-19, or nosocomial infections before and after the epidemic of COVID-19.

### Statistical Analysis

The χ^2^ tests were used to compare the prevalence of the symptoms of common mental disorders by characteristics groups. Logistic regressions were performed for each type of mental health problem to evaluate the independent effects of perceived psychological feelings controlled by hospitals and participants characteristics. *P*-values < 0.05 were considered statistically significant in this paper. All analysis was implemented in SAS 9.4.

### Elastic Net Regularization

Following Marcon et al. (19) method in dealing with the bias-variance trade-off in multiple regressions, a regularization technique called the elastic net regularization was applied to reduce inflating variance stemming from both the inclusion of many predictors in the linear model and highly correlated predictors of the symptoms of common mental disorders at the cost of introducing some bias. The elastic net is a machine learning method that convexly combines ridge regression which penalizes sum of squared coefficients (*l2* penalty), and LASSO (least absolute shrinkage and selection operator) regression which penalizes the sum of absolute values of the coefficients (*l1* penalty). As both individual-level and hospital-level characteristics were considered to be potential predictors of mental health problems, the predictive performance of the model was expected to be increased by minimizing model's error at the optimal model complexity.

Specifically, data were split into a training (75%) dataset and a test (25%) dataset. An elastic-net regularized generalized linear model (R package *glmnet*, version 3.0-2) was fitted to the training dataset. The resampling method to tune the model was specified as repeated 10-fold cross-validation with 10 repetitions and resulting optimal tuning parameters of the model were then determined. The predicted probability of having symptoms of common mental disorders was obtained by applying estimated modeled parameters to the test dataset. To evaluate the predictive performance of the model, a receiver operating characteristics (ROC) curve was created and the area under the curve (AUC) was calculated. All analysis was implemented by R software (version R 4.1.0), and all training process was realized in the R package *caret* (version 6.0-86).

## Results

### Sample Description

A total of 4,677 doctors (39.6%) and nurses (60.4%) from 348 hospitals in 31 provinces of mainland China completed the online survey from February 17 to 24, 2020. The average age for the sample was 35.9 ± 9.0 years old, with its majority being female medical staff (82.3%). Totally 44.2% of the participants were frontline medical staff who worked at departments with high-risk exposure to COVID-19. Main outcome measures, including psychological distress, depressive symptoms, and anxious symptoms, were available among all 4,677 medical staff.

### Occupations and the Symptoms of Common Mental Disorders

The prevalence of psychological distress, anxious symptoms, and depressive symptoms was 15.9% (95% CI 14.8–16.9), 16.0% (95% CI 15.0–17.1), and 34.6% (95% CI 33.2–35.9), respectively. Frontline doctors and nurses had higher prevalence of the symptoms of common mental disorders compared with those who worked at departments with low-risk exposures. Specifically, compared with doctors working at low-risk departments, frontline doctors had higher prevalence of psychological distress (21.7 vs. 12.9%, χ^2^ = 25.50, *p* < 0.001), anxious symptoms (17.1 vs. 11.0%, χ^2^ = 14.44, *p* < 0.001), and depressive symptoms (32.0 vs. 23.8%, χ^2^ = 15.42, *p* < 0.001). Similar trends were observed for nurses. The prevalence of psychological distress (17.7 vs. 13.3%, χ^2^ = 10.54, p = 0.001) and anxious symptoms (19.7 vs. 15.9%, χ^2^ = 6.88, *p* = 0.009) differed across departments, where frontline nurses had higher prevalence than nurses working at low-risk departments did. For frontline medical staff, nurses had higher prevalence of depressive symptoms (40.6 vs. 32.0%, χ^2^ = 15.72, *p* < 0.001) and lower prevalence of psychological distress (17.7 vs. 21.7%, χ^2^ = 4.98, *p* = 0.026) than doctors did. For medical staff working at low-risk departments, nurses had higher prevalence of depressive symptoms (38.0 vs. 23.8%, χ^2^ = 56.83, *p* < 0.001) and anxious symptoms (15.9 vs. 11.0%, χ^2^ = 12.38, *p* < 0.001) than doctors did.

### Perceived Psychological Feelings and the Symptoms of Common Mental Disorders

As demonstrated in Table 1, all perceived psychological feelings were significantly associated with the symptoms of common mental disorders (*p* < 0.001). Medical staff who had higher level of the following feelings, including overloaded work, not having enough time to rest, feeling to have physical or psychological problems in the past, not having adequate protective equipment while working, weak care from directors, and lack of support from colleagues or family members, had higher prevalence of the symptoms of common mental disorders than those without or having lower level of these feelings.

**Table 1:** Prevalence of three psychological problems by perceived psychological feelings of the sample.

**Perceived psychological feelings**	**Groups**	***N***	**Psychological distress**	**Anxious symptom**	**Depressive symptom**
			**% (95% CI)**	**% (95% CI)**	**% (95% CI)**
Overloaded work	No	3,408	8.9 (7.9–9.8)	10.0 (9.0–11.0)	28.3 (26.8–29.9)
	Yes	1,269	34.7 (32.1–37.3)	32.2 (29.7–34.8)	51.2 (48.5–54.0)
			*p* < 0.001	*p* < 0.001	*p* < 0.001
Not having enough time to rest	No	3,393	9.3 (8.3–10.2)	9.7 (8.7–10.7)	27.2 (25.7–28.7)
	Yes	1,284	33.3 (30.8–35.9)	32.7 (30.1–35.3)	54.0 (51.3–56.8)
			*p* < 0.001	*p* < 0.001	*p* < 0.001
Previous physical health conditions	Very good	1,743	6.1 (5.0–7.2)	9.0 (7.6–10.3)	25.0 (23.0–27.0)
	Good	1,932	15.0 (13.4–16.6)	14.2 (12.6–15.7)	33.0 (30.9–35.1)
	Not very good	1,002	34.5 (31.6–37.5)	31.9 (29.0–34.8)	54.1 (51.0–57.2)
			*p* < 0.001	*p* < 0.001	*p* < 0.001
Previous mental health conditions	Very good	1,967	6.5 (5.4–7.6)	8.2 (7.0–9.5)	23.6 (21.8–25.5)
	Good	1,874	15.0 (13.4–16.7)	14.6 (13.0–16.2)	32.9 (30.8–35.1)
	Not very good	836	39.7 (36.4–43.0)	37.6 (34.3–40.8)	63.9 (60.6–67.1)
			*p* < 0.001	*p* < 0.001	*p* < 0.001
Protective equipment	Adequate	2,925	11.6 (10.5–12.8)	12.7 (11.5–13.9)	29.7 (28.1–31.4)
while working	Barely enough	1,333	19.1 (16.9–21.2)	18.8 (16.7–20.9)	38.1 (35.5–40.7)
	Inadequate	419	35.3 (30.7–39.9)	30.8 (26.4–35.2)	56.8 (52.1–61.5)
			*p* < 0.001	*p* < 0.001	*p* < 0.001
Care from directors	Strong	2,787	9.3 (8.2–10.4)	10.7 (9.6–11.9)	27.4 (25.7–29.0)
	Fair	1,820	24.0 (22.0–26.0)	22.5 (20.6–24.4)	44.2 (41.9–46.5)
	Weak	70	65.7 (54.6–76.8)	58.6 (47.0–70.1)	70.0 (59.3–80.7)
			*p* < 0.001	*p* < 0.001	*p* < 0.001
Support from colleagues	Strong	2,282	6.7 (5.7–7.8)	8.6 (7.5–9.8)	24.7 (22.9–26.5)
	Fair	2,285	22.9 (21.2–24.6)	21.4 (19.7–23.1)	42.4 (40.4–44.4)
	Weak	110	59.1 (49.9–68.3)	58.2 (49.0–67.4)	75.5 (67.4–83.5)
			*p* < 0.001	*p* < 0.001	*p* < 0.001
Support from family members	Strong	2,896	9.9 (8.8–11.0)	10.5 (9.4–11.7)	27.6 (26.0–29.2)
	Fair	1,431	20.9 (18.8–23.0)	21.1 (19.0–23.2)	41.2 (38.7–43.8)
	Weak	350	44.9 (39.6–50.1)	40.9 (35.7–46.0)	64.9 (59.9–69.9)
			*p* < 0.001	*p* < 0.001	*p* < 0.001

### Multivariate Logistic Regressions of the Symptoms of Common Mental Disorders

Results from multivariate logistic regressions are shown in Table 2. After adjusting for hospitals and participants characteristics, all of the negative psychological feelings, including feeling overload of work, not having enough time to rest, not having adequate protective equipment while working, weak care from directors, lack of supports from colleagues or family members, and feeling not very good of physical or mental health conditions in the past, were risk factors of psychological distress, anxious symptoms, and depressive symptoms. The feeling of less support from colleagues had the strongest correlations among all psychological feelings in three models, including psychological distress (OR = 3.3, 95% CI: 2.0–5.6), anxious symptoms (OR = 3.3, 95% CI: 2.0–5.4), and having all three problems (OR = 4.8, 95% CI: 2.7–8.5). The feeling of having psychological problems in the past had the strongest correlation with depressive symptom (OR = 3.4, 95% CI: 2.6–4.3) among all perceived psychological feelings.

**Table 2:** Multivariate logistic analysis for the correlations of perceived psychological feelings with three psychological problems.

**Perceived psychological feelings**	**Groups**	**Psychological distress OR (95% CI)**	**Anxious symptom OR (95% CI)**	**Depressive symptom OR (95% CI)**	**All three psychological problems OR (95% CI)**
Overloaded work	No	1	1	1	1
	Yes	2.3 (1.8–2.9)	1.9 (1.5–2.4)	1.3 (1.1–1.6)	2.2 (1.6–2.9)
Not having enough time to rest	No	1	1	1	1
	Yes	1.8 (1.5–2.3)	2.2 (1.8–2.7)	2.0 (1.7–2.4)	2.3 (1.8–3.1)
Protective equipment	Adequate	1	1	1	1
while working	Barely enough	1.0 (0.8–1.3)	0.9 (0.8–1.2)	1.0 (0.8–1.1)	1.1 (0.8–1.4)
	Inadequate	1.7 (1.3–2.2)	1.2 (0.9–1.6)	1.5 (1.2–2.0)	1.7 (1.2–2.4)
Care from directors	Strong	1	1	1	1
	Fair	1.0 (0.8–1.3)	1.0 (0.8–1.2)	1.2 (0.996–1.4)	1.2 (0.9–1.6)
	Weak	3.0 (1.6–5.7)	2.2 (1.2–4.0)	1.6 (0.9–2.9)	3.1 (1.7–5.9)
Support from colleagues	Strong	1	1	1	1
	Fair	1.8 (1.4–2.3)	1.4 (1.1–1.8)	1.2 (1.04–1.5)	1.9 (1.4–2.7)
	Weak	3.3 (2.0–5.6)	3.3 (2.0–5.4)	2.7 (1.6–4.5)	4.8 (2.7–8.5)
Support from family members	Strong	1	1	1	1
	Fair	1.3 (1.01–1.5)	1.4 (1.2–1.7)	1.2 (1.03–1.4)	1.4 (1.04–1.8)
	Weak	2.4 (1.8–3.3)	2.2 (1.6–3.0)	2.0 (1.6–2.7)	2.3 (1.6–3.3)
Previous physical health conditions	Very good	1	1	1	1
	Good	1.6 (1.2–2.2)	0.9 (0.7–1.2)	0.9 (0.8–1.2)	1.5 (0.998–2.3)
	Not very good	2.3 (1.7–3.2)	1.3 (0.9–1.7)	1.2 (0.9–1.5)	1.9 (1.2–2.9)
Previous mental health conditions	Very good	1	1	1	1
	Good	1.4 (1.02–1.8)	1.4 (1.05–1.8)	1.3 (1.1–1.6)	1.3 (0.9–2.0)
	Not very good	3.1 (2.2–4.2)	3.1 (2.3–4.2)	3.4 (2.6–4.3)	3.6 (2.4–5.4)

### Results From Elastic Net Regularizations

**Figure 1:**
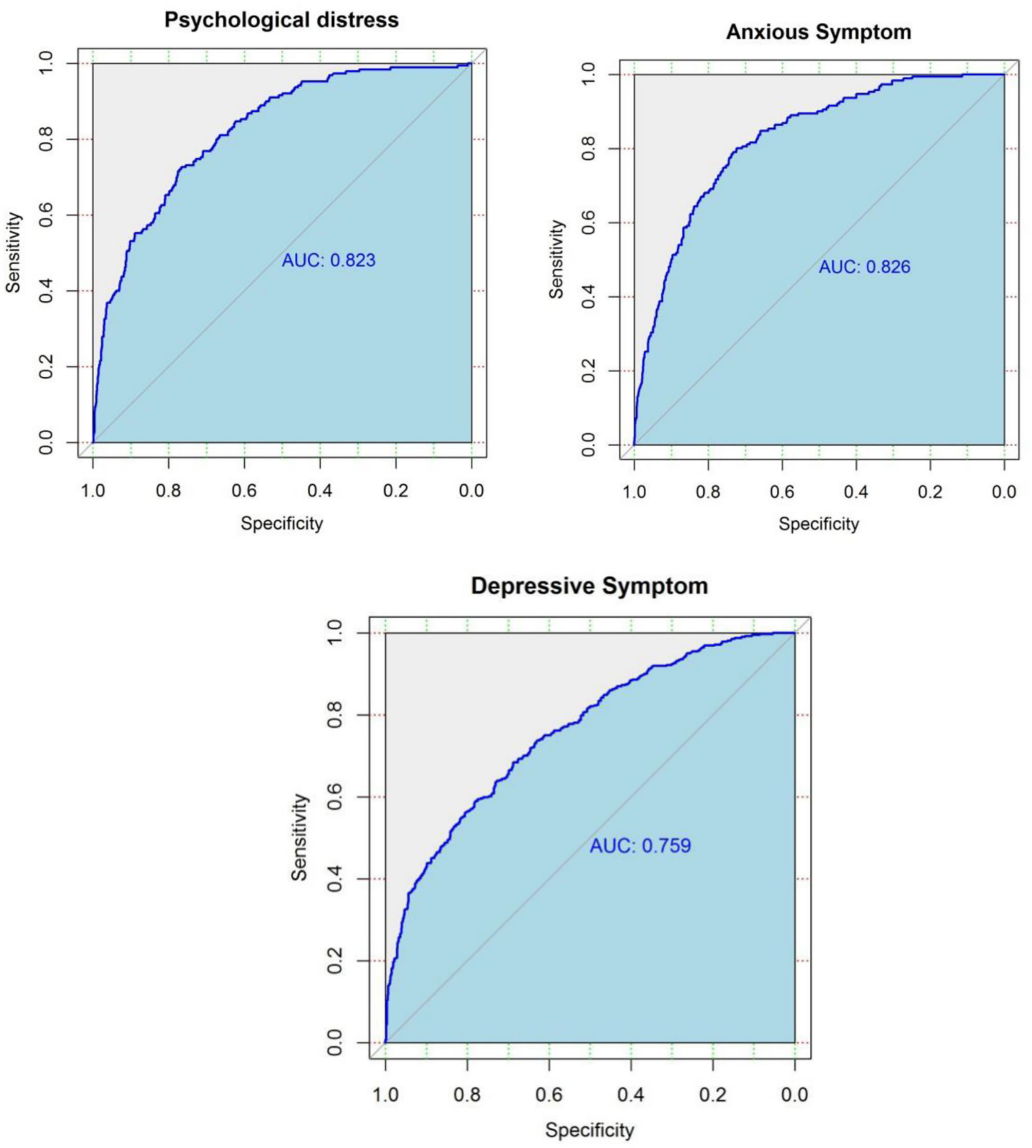
Receiver operating characteristic (ROC) curves for predicted probability of the symptoms of common mental disorders among medical staff based on elastic net algorithm in the test dataset (25% of the whole sample).

**Figure 2:**
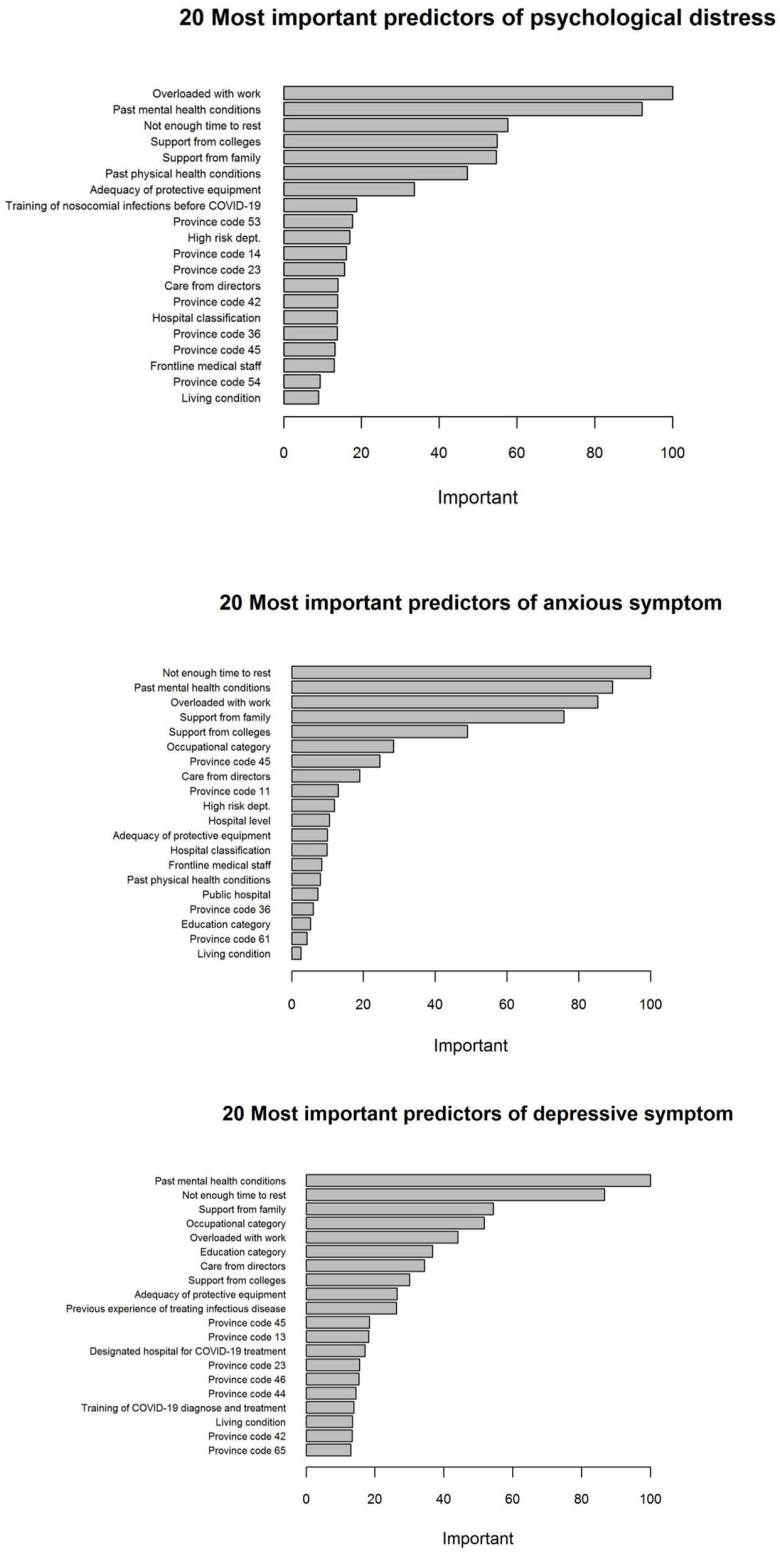
Twenty most important predictors of the symptoms of common mental disorders based on the Elastic Net models.

The ROC curves from elastic net models are presented in Figure 1. An AUC of 0.82, 0.83, and 0.76 was found for models predicting psychological distress, anxious symptoms, and depressive symptoms, respectively, showing a relatively good capability of distinguishing between respondents with mental health symptoms and no symptom. Figure 2 demonstrates the most important predictors in Elastic Net models. The feelings of overloaded work, previous mental health conditions, not having enough time to rest, and lack of support from family members or colleagues were the variables with highest weights in predicting the symptoms of common mental disorders.

## Discussion

### Main Findings and Interpretations

This study is a large sample survey of the symptoms of common mental disorders among medical staff during the early stage of the epidemic of COVID-19 in China. At that time, the epidemic has not been controlled. Medical staff had to face huge work pressure and were exposed to the risk of infections. Although the importance of psychological aids or interventions had been widely recognized, systematic strategies of psychological interventions had not been developed to increase the mental health status of medical staff (20, 21). Therefore, the aims of this study were to identify prior population for psychological interventions and to understand the contributions of psychological feelings to the symptoms of common mental disorders, so as to identify the mandates during the psychotherapies.

From the survey, it is clear that both doctors and nurses working at high-risk departments tended to have more symptoms of common mental disorders than those working at low-risk departments did. These results suggest priority should be given for frontline medical staff when mental health professionals provide psychological aids or interventions during the epidemic of COVID-19. This finding was consistent with the results from studies conducted during the outbreak of Severe Acute Respiratory Syndrome (SARS). It was reported that medical staff in high-risk situation, such as working at SARS units, tended to have higher risk of psychological problems compared with those in low-risk situation, and working as nurses had poorer outcome than that of doctors (22). Furthermore, high-risk exposure could also explain the reasons why another Chinese survey on medical staff (23) reported higher prevalence of psychological problems than our findings. In that study, most samples were from Wuhan (60.5%) or other cities in Hubei province (20.8%), where the risk of exposure was relatively higher than that in other areas in China. Results also showed that the patterns of symptoms of common mental disorders were different between doctors and nurses. Nurses tended to have more depressive and anxious symptoms. This might be related to the fact that nurses usually had higher workload than that of doctors. They need to provide direct care for COVID-19 patients and stay longer in wards, so their risk of exposure is higher than doctors. Moreover, their mental health status may also be influenced by conditions or psychological problems of patients (23). This finding suggests more attention should be paid to nurses during the psychological aids or interventions. Earlier studies pointed out that nurses might be more likely to have seasonal affective disorder (SAD) than other mental health staff did (24), and seasonal sensitivity was more remarkable among female than that in male (25). However, current evidences showed different patterns of SAD across countries (26). Winter SAD was more likely to be reported in western countries, such as the United States (27), while summer SAD was usually seen in eastern countries, such as China (28) and Japan (29). Therefore, although the data collection of this study was carried out during the winter time, it is unlikely that winter season had a contribution to depressive symptoms among this sample. Nevertheless, more researches need to be carried out in this field to understand the relationship between seasons and affective disorder during the epidemic of COVID-19.

Psychological aids or interventions are helpful to decrease the psychological problems during or after a crisis event (30–32). A satisfied psychological intervention should be carried out based on clear identification of therapeutic mandates (33). In this study, perceived psychological feelings were collected to explore the potential therapeutic mandates of medical staff. Three types of perceived psychological feelings, including workload, social support, and perceived health status, were reported by medical staff. Multivariate regression results confirmed that aforementioned perceived psychological feelings had significant correlations with the symptoms of common mental disorders, which coincided with the findings from the elastic net models. Feelings of overloaded work and insufficient rest time, and previous experiences of psychological problems had the largest contributions to the prediction of psychological distress and anxious symptom. Feelings of supports from family members or colleagues were also important predictors. In the elastic net model of predicting depressive symptom, similar patterns were identified, with greater weights on supports from family members and colleagues.

The above findings confirm that the urgent demand of medical staff with psychological problems are the alleviation from job stress and having enough rest (34, 35). Significant increase of work stress and lack of rest are common issues confronted by medical staff. When an epidemic outbreak occurs, overloaded with work would lead to psychological problems of medical staff, as the case of SARS outbreak (36). During the COVID-19 pandemic, stress at work mainly stems from the concerns of being infected and the unknowns to the disease. Like Ebola, Middle East Respiratory Syndrome (MERS), SARS, and other newly identified infectious diseases, there is no specific antiviral treatment for COVID-19. Moreover, COVID-19 symptoms develop so fast that it could lead to multiple organ failure and even sudden death. Medical staff's timely judgements of the conditions of patients and supportive cares become the major treatment of COVID-19. Consequently, work stress upon medical staff when treating COVID-19 patients are much higher than that in daily work. During the COVID-19 pandemic, medical staff is overwhelmed by COVID-19 patients. Long time working in the COVID-19 wards wearing PPE makes job burnout an unavoidable problem faced by all medical staff. Previous studies have found a negative relationship with work stress and positive mental health, among which job burnout plays as a crucial mediator (37). Reduced level occupational stress was found to suppress the development of symptoms of mental disorders (38). Therefore, when providing psychological aids or interventions to medical staff, strategies could be discussed with these staff to cope with work stress, limit the work hours, enhance training, and increase resting time. To achieve better effects of the intervention, the measures to conduct the interventions should also be concerned (39), and different plans might be applied in different stage of the epidemic (40).

Additionally, the results of this study showed that previous worse mental health conditions were highly correlated with current symptoms of common mental disorders under stressful event, which are in line with findings from other researches (41, 42). The explanation would be that medical staff with previous worse mental health conditions appeared to be less resilient to the adverse effects of stressful event than their colleagues with better mental health conditions did. Supports from both colleagues and family members were found to be an active coping strategy of overcoming psychological problems and reducing mental health symptoms, which highlight the importance of social support in psychological interventions and mental health improvement under emergent events.

The elastic net model used in this paper provided a more flexible model specification by decreasing model complexity while keeping all potential predictors. Consequently, it increased the model's ability at distinguishing between medical staff with symptoms of common mental disorders and those without, yielding a relatively high AUC that ranges from 0.76 to 0.83. Compared with traditional regression techniques, the elastic net model further presents the relative importance of each predictor, which facilitates the comparisons across variables on the basis of their model contributions. The predicting ability of models in the elastic net framework was further explored. The AUCs from elastic net models range from 0.76 to 0.83, presenting a relative good performance of prediction. Overloaded with work, previous mental health conditions, insufficient time to rest, and support from both family and colleagues were found to be the predictors of greatest importance, which stands out the need of evaluating current and previous mental and physical conditions in predicting mental health symptoms.

## Conclusions

### Strength and Limitation

This paper aims to test the contributions of perceived psychological feelings to the symptoms of common mental disorders among doctors and nurses in hospitals during the epidemic of COVID-19 in China, and to identify the population with highest needs of psychological interventions. This work is one of the pioneer large-sample studies on the symptoms of common mental disorders of medical staff during the early stage of COVID-19 epidemic in China. Results in this study confirm that perceived psychological feelings of workload, social support, and health status make important contributions to the symptoms of common mental disorders of medical staff during the COVID-19. Strategies of psychological aids or intervention should be developed based on perceived psychological feelings of medical staff with symptoms of common mental disorders. Findings from the survey can help mental health professionals respond to the challenge and implement interventions effectively and efficiently.

Below are the limitations of the study. First, the sample in the study was from a non-probability sampling design, and female respondents were much more than male. That to some extent may influence the representativeness of larger medical staff. However, as the survey was carried out during the epidemic period of COVID-19, a convenient sampling design might be the most feasible choice and could minimize the impact on the work of medical staff. Furthermore, 1-week data collection period was determined in order to increase the homogeneity of the sample and avoid psychological effects by outside environment. Multivariate analysis was carried out to control the potential gender bias. Second, it should be noted that the screening for the symptoms of mental disorders was carried out in the survey. Only possible cases, rather than patients with clear diagnoses, could be identified by the self-reported instruments. It was also difficult to understand the effect on mental health due to the epidemic, as the histories of physical or mental disorders were not collected. Therefore, comprehensive evaluations should be emphasized before any psychological interventions or psychiatric treatments.

### Implications

Perceived psychological feelings have significant correlations with the symptoms of common mental disorders among medical staff during the epidemic of COVID-19. When mental health professionals provide psychological aids or interventions for medical staff with psychological problems during the epidemic, reducing the work intensity, increasing the social support especially from family members and colleagues, and improving previous mental health status could be suggested to these staff, and priority should be given to frontline medical staff and nurses.

## Data Availability Statement

The raw data supporting the conclusions of this article will be made available by the authors, without undue reservation.

## Ethics Statement

The studies involving human participants were reviewed and approved by the Ethical Committee of Beijing Ditan Hospital Capital Medical University (JINGDILUNKE(2020)-(012)-01). Written informed consent for participation was not required for this study in accordance with the national legislation and the institutional requirements.

## Author Contributions

RJ is one of the members of National COVID-19 Expert Group and led the research team. BH acted as research coordinator and was responsible for data collection with assistance of JW and YW. ZL and RJ designed the study. BH and CM wrote the first draft. CM, TZ, and HC did the analyses, with supervision from ZL and YH. All authors reviewed the report, provided further contributions and suggestions, read, and approved the final report.

## Conflict of Interest

The authors declare that the research was conducted in the absence of any commercial or financial relationships that could be construed as a potential conflict of interest.

## Publisher's Note

All claims expressed in this article are solely those of the authors and do not necessarily represent those of their affiliated organizations, or those of the publisher, the editors and the reviewers. Any product that may be evaluated in this article, or claim that may be made by its manufacturer, is not guaranteed or endorsed by the publisher.
